# Experimental Researches Applied to Physiology and Pathology

**Published:** 1852-09

**Authors:** E. Brown-Séquard

**Affiliations:** Paris


### MEDICAL EXAMINER,

AND

### RECORD OF MEDICAL SCIENCE.

NEW SERIES.—NO. XCIII.—SEPT., 1852.

---

### ORIGINAL COMMUNICATIONS.

---

### *Experimental Researches applied to Physiology and Pathology.*

By E. Brown-Séquard, M. D., of Paris.

(Continued.)

VII.—ON THE CAUSES OF THE TORPIDITY OF THE TENREC, (*Erinaceus ecaudatus*, Linn.)

The influence of cold as a cause of hybernation has not been considered essential, on account of an assumed fact, which is altogether incorrect. This fact is that a mammal called the Tenrec, (*Erinaceus ecaudatus*, Lin.,) which lives in the Islands of Mauritius, of Madagascar and Bourbon, is torpid under the joined influences of warmth and dryness. Cuvier says, about the Tenrec: "They are nocturnal animals, remaining in lethargy three months each year, although they inhabit the torrid zone. Moreover, Bruguiere asserts that it is during the time of the greatest heat that they are torpid." Physiologists have admitted this fact as true, and they have supposed that warmth and dryness could be, like cold, a cause of torpidity.

The following facts prove that the torpid sleep of the tenrec takes place from precisely the same cause as that of the hedge-hog, (*Erinaceus Europeus*, Lin.,) and of other hybernating mammals:

1st. The tenrec and the hedge-hog belong to the same family, and they are much alike.

2d. According to MM. Desjardins, Telfair and Coquerel, and to my own observations, the tenrecs earth themselves, and are torpid from the month of June to the month of November, *i. e.* during the winter season of the Islands where they live.

3d. Hybernating animals, belonging to varied species, observed by Pallas, Mangili, Marshall Hall, Berthold, Barkow, and myself, have been found torpid at the temperature of 61 to 66° F., (16 to 19° Cs.) Moreover, I have found that dormice, (*Mus glis.*, Lin.) even at a temperature of 68 to 72° F., (20 to 22° Cs.) may become torpid, and I have observed some that were constantly sleeping, during a whole week, at a temperature varying from 59 to 68° F. (15 to 20° Cs.) I have stated that hedge-hogs may be torpid during the summer; in Paris, at a temperature of 68 to 72° F., (20 to 22° Cs.) and lately, in Philadelphia, I have seen a marmot (*Arctomys monax*, Buff.) torpid, in June, at a temperature of 71 to 73° F. (21.5 to 23° Cs.)

4th. During the time of its torpidity, the tenrec is under the influence of a temperature of 59 to 72 or 73° F., (15 to 22 or 23° Cs.) rarely more, and sometimes less. Therefore these animals are exposed to a temperature sufficiently low to render them torpid, for that temperature may produce that effect on the hybernating animals of cold countries.

From these facts I believe it is right to conclude that torpidity is induced in the tenrec, in the same way as in the other hybernating mammals, and therefore it is not necessary to suppose that warmth and dryness produce torpidity.

### VIII.—ON THE INFLUENCE OF POISONS UPON ANIMAL HEAT AS A CAUSE OF DEATH.

Prévost and Chossat, and after them, M. Magendie, have ascertained that death occurs quickly in mammals when their temperature is notably diminished. My experiments confirm the correctness of that statement. The diminution of animal heat in mammals, is so dangerous that, in one case, I have seen death take place in a rabbit after a diminution of only 22° F. (12° Cs.) I have never observed any animal continuing to live when I had diminished its temperature more than 44° F. (24°.5 Cs.) I have found the law established by Chossat perfectly correct, according to which the diminution of animal heat necessary for killing is less and less, in proportion to the rapidity with which that diminution takes place.

It is very probable that in all the cases where, in consequence either of disease, or of a wound, or of poison, the temperature of man is diminished many degrees, his life is in danger from the very fact of that diminution. It is thus in cholera, in sclerema, in certain cases of palsy, in cases of great disturbance of the respiration, in fractures and luxations of the vertebral column, in the cervical, and even in the dorsal regions, in cases of profuse hæmorrhage, and in many cases of poisoning when death is not rapidly produced.

It has been long known that temperature is diminished in poisoned persons; and there are but few cases of poisoning on record in which it is not said that the patient was cold. Chossat has found that a dog, into whose veins he had injected opium, had its temperature diminished from 105° to 62.6° F. (40°.3 to 17° Cs.), 22 hours after the injection. Brodie has found that many poisons act upon animal heat so as to diminish it considerably. Demarquay and Duméril, junior, and later, these two experimenters, joined with Lecointre, have found the same thing as Brodie in many toxic agents. I have made very numerous experiments on this subject, and some of their results have been published before the last papers of Demarquay, Duméril and Lecointre.*

I have stated that many poisons, either injected in the veins or absorbed by the vessels of the skin or of the digestive canal, may diminish sufficiently the temperature of Guinea pigs and rabbits, to produce death. This occurs when the dose of the poison is not large enough to kill in less than four or five hours. These poisons may kill only by their action upon

* See Gaz. Med. de Paris, 1849, t. iv., p. 644.

animal heat. It may be so with opium, cyanhydric acid, the cyanide of mercury, hyoscyamus, digitalis, belladonna, tobacco, euphorbia, camphor, alcohol, acetic, oxalic, sulphuric, azotic, chlorohydric acids much diluted, and some oxalates.

Of course the action of these poisons is the greater, the colder the atmosphere; but it is not always immediately so, and instead of diminishing the animal heat, many may increase it for a time, especially when the temperature of the atmosphere is elevated.

I have discovered that a dose of one of these poisons, sufficient to kill an animal, when there is no obstacle to the diminution of its temperature, may be unable to destroy life when the temperature of the animal is maintained by artificial means to its normal degree, or not far from it. My experiments have been conducted as follows:

Equal doses of poison were given, simultaneously, to two animals, as much alike one another as possible. One of them was left in a room at a temperature of from 46 to 50° F. (8 to 10° Cs.), and the other was kept not far from a chimney, in a place where the air was at from 75 to 86° F. (24 to 30° Cs.) The first was dead after a certain number of hours, or sometimes one or two days, having its temperature much diminished. The other, on the contrary, had no perceptible diminution of its temperature, and was generally cured very soon. Therefore, when taken in certain doses, many poisons may kill only by their influence on animal heat, and physicians, in cases of poisoning, should try as much to prevent the diminution of temperature, as to expel the poison or to act against it by an antidote, by pulmonary insufflation, or otherwise.

In experiments which I have made lately on the action of very pure digitaline, that had been prepared by M. Quévenne, the celebrated chemist, who has made such interesting and accurate researches on digitalis and the substances of which it is composed, I have found that this poison may also diminish temperature. I believe it is easy to explain the contradiction existing between Traube and Stannius, as regards the influence of digitalis on animal heat. When the atmosphere, in which the animal is, is cold, then its temperature may be diminished by digitalis or digitaline, but when it is warm the diminution does not take place, or it is very small. But, of course, if the dose is sufficient to kill very quickly, then it is indifferent whether the atmosphere is cold or not, because there may be not time enough for the diminution of the temperature of the animal.

I have to relate another fact which, I believe, ought to be considered as analogous to the preceding. It is that kind of poisoning which occurs when a layer of oil, of varnish or of gelatin, is put on the skin of a warm-blooded animal. Death, then, is very probably produced by a substance unknown until now, and which is secreted by the skin. The layer of oil, varnish, or gelatin preventing that secretion taking place, that unknown substance becomes accumulated in the blood, and then are produced the phenomena so well studied by MM. Fourcault, Becquerel, Breschet, and Magendie. I have found that in such a case the animals may live, if the atmosphere in which they are kept is at a temperature inferior to 79 or 80° F. (26 or 27° Cs.) In these circumstances their temperature is not sensibly diminished, while it diminishes much when the atmosphere is cold. Therefore it is especially by their loss of warmth that animals are killed, when their body has been entirely covered with oil, varnish, or gelatin.

### IX.—On certain actions of cold, warmth, and light upon the crystalline lens.

Pourfour du Petit has discovered that after the death of a mammal, it is not uncommon to find the crystalline lens opaque. He has found also, that when the lens has become opaque, if we draw it near the flame of a candle or a lamp, it becomes transparent again after a few minutes. The same lens, put alternately near and far from the flame, may become alternately transparent and opaque. I have endeavored to discover whether it is the light or the warmth of the flame which renders an opaque lens transparent. I have placed between the lens and the flame a layer of mineral salt, which is athermane and transparent. Then the light of the flame could reach the lens, but not its warmth. There has been no action. In another experiment I have exposed to the light passing through the mineral salt a fresh crystalline lens, still perfectly transparent. It became opaque. Now, in a third experiment, I have compared two crystalline lenses, perfectly transparent, one exposed to the action of light passing through the mineral salt, and the other kept in an obscure place. The former became opaque much quicker, and considerably more than the other. Therefore, 1st. It is not light which renders transparent an opaque lens; 2d. Light does not prevent a transparent lens from becoming opaque; 3d. Moreover, light appears to accelerate, if not to produce, the opacification of the crystalline lens.

As to the influence of warmth, it is certain that it is that which renders transparent an opaque lens: 1st. When left in the atmosphere, at a temperature superior to 70° F. (21° Cs.), a fresh transparent lens remains transparent. 2d. When a lens has become opaque, it is frequently sufficient to keep it exposed to the warmth of the hand, during some minutes, to render it transparent. 3d. The lower the temperature of the atmosphere the quicker transparent lenses become opaque.

These experiments give a very interesting result, *i. e.*, that light appears able to produce an effect precisely opposite to the effect produced by warmth.

From these researches I conclude:

1st. That light, and a low temperature, are favorable conditions, if not direct causes, of the opacity of the lens in warm-blooded animals after death.

2d. That a warm temperature, *i. e.*, a temperature superior to 70° F. (21° Cs.) is a cause of the change occurring in the lens, by which, when opaque, it becomes transparent.

I will add, that sometimes an analogous opacity takes place in the cornea.

### X.—ON THE NORMAL DEGREE OF THE TEMPERATURE OF MAN.

The degree of animal heat, in the human species, is stated as being between 98.5° and 100° F. (37 and 38° Cs.) I intend to prove that it is higher.

It is impossible to take directly the temperature of most of the internal parts of the body in man; therefore many physiologists, in order to discover the temperature of these parts, have argued as follows: According to J. Hunter, the temperature of the rectum in animals is the same as that of the right ventricle of the heart; hence it has been concluded that as the temperature of the rectum, in man—still according to Hunter—is 98°.42 F. (36°.9 Cs.) the temperature of the internal parts of the body ought to be between 98 and 99° F. (36°.67 and 37°.22 Cs.)

Some other physiologists, noting the temperature of the mouth under the tongue, and supposing that this temperature is nearly the same as that of the internal parts of the body, have concluded that the temperature of man is between 99 and 100° F. (37°.2 and 37°.8 Cs.)

We will prove that these deductions are not right:

Firstly, the degree of the temperature of the rectum is not, as Hunter says, 98°.4. It may be so in debilitated men, but in healthy persons it is more elevated. It is between 100 and 102° F. (37.7 and 38°.89 Cs.) according to my own researches and to those of Berger and Maunoir. Besides, many experiments on dogs, rabbits and guinea-pigs, have shown satisfactorily to myself that the temperature of the rectum is not equal to that of the right ventricle. This last organ is from 1 to 3° F. (0.56 to 1.7° Cs.) higher than the rectum.

The temperature of the rectum, in man, being between 100 and 102° F. (37°.7 and 38°.8 Cs.), and the temperature of the right ventricle, in man, being from 1 to 3° F. (0.56 to 1.7 Cs.) higher than that of the rectum, if we suppose the same difference existing in man as in mammals, it follows that the temperature of the right ventricle of the heart in man ought to be between 101 and 105° F. (38°.33 and 40°.56 Cs.)

But a closer approximation of the exact temperature of the internal parts of the body, in man, may be obtained by taking the temperature of an organ situated deeper than the rectum and, consequently, less exposed to the influence of the atmosphere. Such is the case in the bladder. I have observed its temperature by taking that of the urine at the moment of its emission and before any sensible change had occurred in it. The urine was directly received in a vase dipped into a large quantity of water at 98° F. (36°.7 Cs.) Thus I have ascertained that the mean temperature of the urine, in man, is 102°.6 F. (39°.2 Cs.)

Now if we take notice of this well-known fact, that the temperature of the lower part of the abdomen is less elevated than the upper part, we are authorized to believe that the temperature of the central parts of the body, in man, is very near 103° F. (39°.5 Cs.)

My experiments on the temperature of urine were made on ten strong sailors, in the spring, on the Atlantic Ocean, between the 43d and 45th deg. of north latitude. The lowest degree of the temperature of urine which I have observed, was 100°.9 F. (38°.3 Cs.); the highest was 103°.2 F. (39°.56 Cs.) My own urine, examined more than thirty times, in the most varied conditions, has been nearly always at the same temperature; the variations have been only between 102° and 102°.8 F. (38°.89 and 39°33 Cs.) The ordinary degree is 102°.5 F. (39°.17 Cs.)

Long before my researches, the temperature of urine, in the human species, had been taken by some observers, but in general without sufficient care. The temperature of the urine is 94°.25, according to Braun; 98°.9, according to De Lisle; and 103°, according to Hales. Recently Berger has taken the temperature of urine in the bladder in five women. He has found it equal to 101°.48 F. (38°.6 Cs.)

As the temperature of woman is a little inferior to that of man, the result obtained by Berger is in accordance with mine.

If we take the average between the temperature of the urine of man as I have found it, and that of woman as Berger has found it, we have a number very near 102° F. (38°.9 Cs.)

From these facts we draw the conclusion that the temperature of the thoracic and abdominal viscera, in the human species and in both sexes, is between 102 and 103° F. (38°.89 and 39°.44 Cs.), *i. e.*, some degrees higher than it is generally admitted.

### XI.—ON THE INFLUENCE EXERTED UPON THE GENERAL TEMPERATURE OF THE BODY BY A CHANGE IN THE TEMPERATURE OF ONE OF THE EXTREMITIES.

The following sentence is given, as an axiom, by Dr. W. F. Edwards: "We cannot either raise or lower the temperature of any one part of the body, without all the other parts of the frame being affected and suffering a corresponding rise or fall in temperature, more or less, according to circumstances."*

* Article *Animal Heat*, in Todd's Cyclop. of Anat. and Physiol., 1839, t. ii., p. 660.

Expressed in such terms, we accept this law as perfectly true. But the author elsewhere gives an extension to this law, which we will prove to be incorrect. No doubt when the temperature of the blood, coming to the heart from a remote part of the body, has been modified in that part, the thoracic viscera ought to have their temperature modified; but to what extent? It is on this very important point that we do not agree with Dr. Edwards. He was of opinion that the influence exerted by a small part, on all the other parts of the body, was considerable. He says that the chilling of a single part, such as the hand or the foot, may cause a loss of temperature in all the other parts of the frame, even far beyond what could have been presumed as likely or possible, and that in a number of experiments where one hand was plunged in water cooled down by ice, the other hand, which was not subjected to the action of the cold bath, lost nearly 5° R. (11°.25 F., 6°.25 Cs.) in temperature.

It will be easily understood how important it would be that practitioners should be able to act on the general temperature of a patient, so as to increase or diminish it, by means of a hand or a foot bath. I regret to say that if the facts observed by Edwards are exact, his conclusion nevertheless is incorrect.

I have performed, both alone, and with the help of Dr. Tholozan, Physician of the Hospital *Val-de-Grace* at Paris, numerous experiments, with the view of knowing whether Dr. Edwards was right or not. I have found that the chilling of one hand plunged in water at the temperature of freezing point, acted very strongly on the temperature of the other hand. But, at first, there is no regularity at all in the quantity of degrees of temperature lost by the hand which remains out of the water; and secondly, we have found once that this hand did not lose any fraction of its temperature. In one case we have observed that the hand kept in the atmosphere did lose 22° F. (12° Cs.) in seven minutes. The ordinary loss of temperature has been of between 6 to 8° F. (3.33 to 4°.44 Cs.) In one case there has been only a loss of 2° F. (1°.2 Cs.) In another case there has been no loss, and, on the contrary, there has been an increase of temperature of 1°.4 F. (0°.8 Cs.)

If, like Edwards, we consider the loss of temperature of the hand not plunged in water as a sign and as a measure of the diminution of the general temperature of the body, we must conclude, from our experiments, that the chilling of a small part of the body may be unable to diminish the temperature of the body sensibly, and that, in other cases, it may act extraordinarily upon it. But the supposition that the hand kept in the atmosphere, was able to give a measure of the modification of the body is incorrect. By taking the temperature of the mouth during the time that one hand was dipped into very cold water, Dr. Tholozan and myself have ascertained that the temperature of the body does not sensibly change. The greatest diminution of the temperature of the mouth has been nearly 1° F. (0°.6 Cs.), and this only in one case. In that experiment in which the hand not plunged in water lost 22° F. (12° Cs.), the temperature of the mouth was not diminished more than the fifth of a Fahr. degree.

As it is quite certain that the temperature of the body is constantly changing, it is easy to understand why we do not find a small but a constant diminution in the temperature of the mouth when one hand is subjected to a notable chilling. When, under the unknown influences that are constantly modifying the temperature of the body, there is a tendency to increase that temperature, then the tendency to its diminution originating from the chilling of one hand may exist without producing any effect. We have there two causes acting in opposite directions, and if they are equal they annihilate each other. If they are unequal, we perceive only the difference between them. When these two causes act in the same direction, then their efforts are added to one another, and it is probably in a circumstance of that kind that I have once found a diminution of 1° F. (0.°56C.) taking place in the mouth.

We have now to examine how the diminution of the temperature of a hand is produced when the other hand is dipped into cold water. *A priori* it is evident that the chilling of the hand kept in the air exists in consequence, either of the arrival of a cooler blood, or in the diminution in the quantity of blood. That hand being exposed to a cold air (for it is only in such a case that the experiments succeed) loses its temperature by the action of that cold air. At first the blood that arrives in the hand is not cooler, as Edwards had supposed it was. This we prove by the fact that the temperature is but very little changed in the mouth. The supposition then remains that the quantity of blood arriving in the hand is smaller than usual. This may happen by two modes, one of which is that the heart sends less blood, and the other that the blood-vessels of the hand are contracted and prevent, in part, the passage of blood. It is certain that the heart continues perceptibly to send the same quantity of blood. Therefore we are induced to admit that the hand's blood-vessels are contracted. But now what is the cause of that contraction? We will try to show that it is in an action of the nervous system. Every one knows that under the influence of a sensation or of emotion, the hands, and sometimes the feet, become cold. The nervous system, in consequence of that sensation or emotion, acts upon the blood-vessels and excites them to contract. The calibre of the visible vessels is sensibly diminished. The same phenomena take place in the two hands when one is dipped into very cold water. An exceedingly violent pain is felt, the nervous centres are strongly excited, and they act then as under the influence of an emotion. Dr. Tholozan and myself have observed that the greater the pain felt, the more the temperature was diminished in the hand left in the air.

As to the influence of partial heating Dr. Edwards relates the following experiment:

"The hand being immersed in water heated to the temperature of 34° R. (108°.5 F.—42°.5 Cs.) rose one degree of the same scale, and the temperature of other remote parts not immediately exposed to the influence of heat were found to have risen to a corresponding degree."

I have repeated this experiment of Dr. Edwards, and I have found no evident elevation in the temperature of remote parts, as the mouth and the hand, not immersed in water.

I conclude, then from the facts contained in this note, that, in general, the temperature of the body is not sensibly modified by the chilling or the heating of a small part of the frame.

XII.—ACTION OF COLD ON THE COAGULABILITY OF BLOOD, AND PERSISTENCE OF LIFE IN FROGS AFTER LOSING HALF OF THE VENTRICLE OF THE HEART.

Dr. Marchal (de Calvi) and other physiologists, have recently asserted that cold diminishes the quantity of fibrine and consequently the coagulability of blood. I have observed a curious fact, which shows that blood may possess a very great coagulability although exposed to the action of cold. I have found that after the removal of the half of a ventricle, in frogs, the blood may coagulate at the surface of the wound and the animal may continue to live. After the mutilation has been made, the lips of the wound are drawn upwards and inside in consequence of the muscular contraction. The blood flows very abundantly, but its coagulation quickly begins, and a layer of solidified blood is soon formed on the entire surface of the section, and by this process the wound is rapidly obliterated. The hemorrhage has been frequently stopped in a few minutes.

This experiment is successful only in cold seasons, probably because the batrachia are able to bear much better a loss of blood at a low than at a high temperature. The pulsations of such mutilated hearts continue with regularity and strength, but the impulse given by the remaining of the ventricle ought to be diminished. Nevertheless the circulation of blood is accomplished well enough to allow the animal to live many months.

I have shown to the *Société de Biologie* at Paris, two frogs on which the wound of the heart was cicatrised after fifteen days.*

### XIII.—ON A SINGULAR CASE OF ANIMAL GRAFT.

Every one knows the experiments by which the cock's spurs or many other animal textures have been grafted on the body of an animal, and especially on a cock's comb. I have succeeded in grafting the tail of a young cat on a cock's comb. I performed this experiment in France in 1850.

After having divided the tail of a young cat, I made a longitudinal section on a cock's comb, and I united these two parts one to the other, by stitching the cut surface of the cat's tail to the cut surface of the cock's comb. The skin of the cat's tail had been turned a little over itself, so that its internal surface was in contiguity with the cut surface of the cock's comb. Eight days after, I punctured the skin of the tail at a distance from the cock's comb, and blood escaped, so that it was evident that circulation was already established. The tail had been cold during

* See Gaz. Méd. de Paris, 1850, t. v. p. 169.

all the day of the operation, but it became warmer gradually from the second day. The union appeared much advanced on the third or fourth day. The tail was entirely fixed on the eighth day.

Unfortunately, on the eleventh day, the cock had a fight with another cock, and the cat's tail was torn out from the ground on which it had been fixed. I was thus deprived of the opportunity of knowing what transformations should have taken place in the tail.

By examining it I found that all its tissues were fresh, and that its blood-vessels contained blood.

### XIV.—ON A CONVULSIVE AFFECTION PRODUCED BY CONSIDERABLE INJURIES OF THE SPINAL CORD.

I have discovered that a very violent convulsive affection is the constant result of a considerable wound of the spinal cord, in certain animals. It is more especially in guinea-pigs in whom a transversal section of a lateral half, or a complete section of the spinal cord has been made, that this is the case.* The section was made at the level of one of the last six dorsal vertebræ or the two first lumbar.

When the convulsive fit begins, the muscles of the face and neck are the first which contract. The convulsions occur alternately in all the muscles of the eye, the face, the tongue, the jaws and the neck; so that the cause of these movements ought to act on the parts of the encephalon, from whence the facial, the trigeminal, the hypoglossus, and the motor nerves of the eyes originate. The head of the animal is alternately bent on both sides of the body, and lastly the limbs are agitated in every direction. In the case of a section of the lateral half of the spinal cord, in the lumbar region or at the end of the dorsal region, three limbs only are strongly convulsed; the two anterior and one of the posterior—the one that is on the side of the body opposite to the side of the section of the spinal marrow. The other posterior limb has only very slight movements. When the spinal cord has been entirely divided in the dorsal or lumbar regions, the two anterior limbs only are convulsed, with the muscles of the face and neck. These con-

* See Gaz. Med. de Paris, 1850, t. v. pp. 651, 895.

vulsions may come without any exterior excitation; but it is very easy generally to provoke them by frightening the animal, or by pinching, burning or otherwise exciting it. The part of the body upon which a mechanical excitation acts more powerfully is the skin of the face or the neck. This convulsive affection has a great analogy with epilepsy; but it has also some distinctive features,—for instance, the animals, during the convulsions, do not appear to lose their consciousness, and they frequently cry when they are pinched.

The affection begins generally eight, ten or twelve days after the spinal cord has been wounded. The convulsions then are not very strong, and it is usually four or five weeks after the operation that the fits are violent and easily produced. Three or four months after, the violence and frequency of these convulsions are diminished; yet in two or three instances they have increased in strength and frequency more than a year after the operation. The affection may last a long time: in one case it still existed two years after the operation.

Generally the fits last from five to fifteen minutes. The stronger they are the shorter is their duration. For an hour or two, and sometimes for one or two days after a violent fit, it is impossible to produce another one by any kind of excitation. When a fit takes place a short time after a violent one, it is always weak or very short.

I have made the following experiment on some guinea-pigs having this convulsive affection: I put them in a small box, so that they had but little room to move, and I gave them food in great abundance. They then ate very much and were deprived of exercise, and, in consequence of that mode of living, they had exceedingly frequent convulsions. One of them had a fit nearly every quarter of an hour. These fits sometimes were strong, but they did not last long. The others had fits three or four times every day. I changed their mode of living, and put them all in a large room where they had only a small quantity of food. The influence of this new regimen was nearly immediate; the same day the number of fits was diminished, and in the course of the third week afterwards they had only one or two fits; and before long some of them were cured.

I must relate also a very interesting fact analogous to the preceding. In several guinea-pigs and frogs, which had the spinal cord transversely divided, I have observed that when the animals remained very quiet during many days, instead of the regular reflex movements, they had a violent tetanic convulsion in their posterior limbs, when the skin of these limbs was pinched.

Such a tetanic movement never occurred in these animals when I excited the regular reflex movements every day.

I have no room in this *résumé* to try to explain the facts related; I will do it in a special paper. I will only add that all the readers of this note, who know the views of Dr. Marshall Hall about convulsive diseases, will remark how strongly the facts I have pointed out are confirmatory of these views.

### XV.—ON THE RELATIONS EXISTING BETWEEN THE ORGANISATION OF NERVE TUBES AND THEIR VITAL PROPERTIES.

It is well known that when the nerve tubes are examined in a very fresh state, their contents, the *medulla*, or *white substance of Schwann*, appear pellucid and homogeneous, and of a fluid consistence; but a kind of coagulation soon takes place in that medulla, making it easily distinguishable from the tube itself, solid, grumous and much less transparent. Water has the power of producing very quickly that kind of coagulation of the white substance of Schwann, and the more quickly the warmer it is.

It was interesting to ascertain whether a nerve-fibre keeps or loses its vital properties when its contents have been transformed by coagulation. I have performed two series of experiments in order to solve this question; one on the motor nerves, the other on the nerves of sensibility.

1st. After having amputated the two limbs of a frog, I laid bare a long portion of the sciatic nerve in both. Half an hour afterwards these two nerves were still able to act perfectly well. Then a microscopical examination of some fibres from one of these nerves demonstrated that the coagulation of the medulla had begun, but was not yet complete. Ten minutes later the nerves were still capable of acting, and the microscope showed that the coagulation was complete. A similar experiment repeated a great many times has always furnished the same result.

2d. After having divided the two sciatic nerves, not far from the knee-joint, in a living frog, I have dipped the central part of these nerves into water. After a few minutes, some fibres taken from one of them were beginning to coagulate; yet these nerves possessed their sensibility. Ten minutes after, there was a complete coagulation of the medulla, while the sensibility was very little if at all diminished.

I draw from these facts the conclusion that nerve-fibres are capable of acting nearly as well when their contents are coagulated, as when they are still liquid.

This result appears to be important, inasmuch as it contributes to show that the white substance of Schwann has not a great part in nervous action. Three reasons concur in demonstrating that the essential part, the truly active one—that which possesses the vital properties—is not the white substance of Schwann:

1. We see that the vital properties of the nerve-fibres do not appear altered, although there is a very material change in this substance when it coagulates.

2. If such a change as that which takes place in this substance when it coagulates, existed in a part possessing the vital properties belonging to nerves, there should certainly be a movement or a sensation accompanying it, and yet there is none. This material change is certainly more considerable than the change effected by a slight mechanical or galvanic excitation, which is capable of producing a movement or a sensation.

3. The microscope has already proved that the white substance does not exist in all the nerve-fibres, and that it is wanting, for instance, in the very fine fibres.

Therefore if it be not the white substance of Schwann which is active in the nerves, and as it is not the *cylinder axis* which possesses the vital properties of the nerves, because it is wanting in many fibres, it results that it is the membranous layer, the paries of the nerve-tube, to which these vital properties belong, unless there is another substance, still unknown, and existing in the tubular canal of the nerve-fibre.

### XVI.—ON THE PERSISTENCE OF LIFE IN ANIMALS DEPRIVED OF THEIR MEDULLA OBLONGATA.*

I. There is no part in the nervous system considered as more essential to life than the medulla oblongata. In the last few years many German physiologists have asserted that this nervous centre is the source of the rhythmical movements of the heart. Besides, an eminent physiologist maintains that in the medulla oblongata a small part exists which is the focus of vital power. Moreover, it is certain that the medulla oblongata has a great share in the respiratory movements. Therefore it seemed probable that the ablation of such an organ, even in cold-blooded animals, ought to be speedily followed by death. Such is not, however, the result of that operation; and in favorable conditions, the batrachia, for instance, can live more than four months after the loss of the medulla oblongata. During all that time, these animals, in appearance remain in good health, and I have observed in them the existence of all the following functions and properties:

1. The circulation of blood continues as well as in unmutilated frogs. The beatings of the heart are at first quickened generally during half an hour, an hour or an hour and a half, after the operation; they then return to their normal rhythm, and they are found as regular and vigorous in frogs deprived of their medulla oblongata, for several days or even several months, as in healthy frogs. Sometimes, particularly when the hemorrhage has been considerable, the beatings of the heart become less numerous and less energetic; then the animal dies very quickly, but if it lives, the movements of the heart resume before long their normal rhythm and strength.

2. The pulsations of the four lymphatic hearts take place as in healthy frogs.

3. Digestion seems to be carried on as well and as quickly in frogs without medulla oblongata as in healthy frogs. I have ascertained this fact by introducing pieces of earth-worms into the stomach of these animals, and by studying the changes produced in these aliments during their passage along the digestive canal. Although very slow, chymous transformation, absorption and the production of fæces took place.

* See the Bulletin de la Soc. Philomatique. Paris, 1849, p. 117.

4. The products of the gastric, intestinal, biliary and pancreatic secretions being very useful, if not essential to digestion, it is very probable that these secretions exist.

5. The urinary secretion and also the production of cutaneous and intestinal epithelium, are performed as usual.

6. The pulmonary respiration ceases, but the cutaneous respiration is continued. The absorption of poisons by the skin and by the mucous membranes exists as in healthy frogs.

7. The reflex faculty is energetic, and so much so that the frogs deprived of the medulla oblongata can raise by a reflex action, greater weights than healthy frogs. As reflex movements exist, I need not say that muscles and nerves have kept their vital properties. It is frequently found that the spinal cord, especially in the *rana temporaria*, becomes so excitable that the slightest irritation of the skin is followed by tetanic convulsions.

8. The galvanic current of muscles not only exists in frogs deprived of medulla oblongata, but appears to be stronger.

From these facts it results clearly that frogs, deprived of their medulla oblongata, are in full life. It is so much so that if they are compared to frogs possessing that nervous centre, they resist etherisation longer, and also live longer after the ablation of the heart.

II. The greatest differences exist in the duration of life, after the removal of the medulla oblongata, in animals of different species, as will be seen in the following table, where the maximum duration of life is indicated in sixty different species of animals:

| Classes. | Species. | Duration of life. |
|---|---|---|
| Amphibia. | Salamanders . . . | More than 4 months. |
| | Frogs . . . . . | |
| | Toads . . . . . | 4 to 5 weeks. |
| Reptilia. | Tortoises . . . . | 9 to 10 days. |
| | Snakes . . . . | 6 to 7 days. |
| | Lizards . . . . | 4 to 6 days. |
| Fishes. | Eel . . . . . | 6 days. |
| | Pike, carp, tench, eel pout, barbel | 3 days. |
| | Perch, gudgeon and others . | 25 to 40 hours. |

| Classes. | Species. | Duration of life. |
|---|---|---|
| Birds. | Sparrowhawk (newly born) . | 21 minutes. |
| | Magpie do. . | 19 minutes. |
| | Sparrow do. . | 17 minutes. |
| | Sparrow, yellowhammer, linnet, pigeon, fowl, duck, pintaw, partridge, moor-hen, turtle-dove (adult) | $2\frac{1}{4}$ to 3 minutes. |
| Mammals.* | Dormouse (during hybernation) | 29 hours. |
| | Hedgehog ditto | 23 hours. |
| | Bull-dog (newly born) . . | 46 minutes. |
| | Cat ditto . . | 41 minutes. |
| | Rabbit ditto . . | 34 minutes. |
| | Guinea-pig ditto . . | 6 minutes. |
| | Dormouse and Hedgehog awaken in summer . . . . | 4 minutes. |
| | Cat, rabbit, guinea-pig and dog (adult) | 3 to $3\frac{1}{4}$ minutes. |

The preceding table shows that after the removal of the medulla oblongata, in different species of animals, the duration of life may be reckoned by *months* for batrachia, by *weeks* for some reptilia, by *days* for other reptilia and for fishes, by *hours* for hybernating mammals, and by *minutes* for birds and non-hybernating mammals.

III. After the removal of the medulla oblongata, the most remarkable differences in the duration of life, in different individuals belonging to the same species, may occur in consequence of differences of temperature. The lower the temperature the longer is the duration of life. Thus, the duration of life in frogs may be reckoned by *months* when the temperature is between 32° and 46° F., (0 and 8° Cs.); by *weeks* when it is between 40° and 55° F. (5° and 13° Cs.); by *days* when it is between 50° and 65° F. (10° and 18° Cs. ) by *hours* when it is between 65° and 72° F. (18° and 24° Cs.); and by *minutes* when it is between 86° and 105° F. (30° and 40° Cs.)

In the other cold-blooded vertebrata the differences in the duration of life, after the removal of the medulla oblongata, are not so great as in frogs, but the law is the same. This law exists also for warm-blooded animals, so much so that the differences existing between mammals of different ages and of differ-

* Pulmonary insufflation has been used only for the dormouse and hedgehog during hybernation.

ent species, are to be attributed, in part, to their differences of temperature.

IV. As the principal condition for a long duration of life in cold-blooded vertebrata is a cold atmosphere, and as the vital phenomena taking place in these animals are much diminished when they are exposed to a low temperature, some physiologists have supposed that the persistence of life for many weeks or more, was equivalent, as regards the sum of the vital phenomena, to a duration of some hours in summer, when these phenomena have a great activity.

In answer to this objection I will at first call the reader's attention to the fact that, in batrachia, deprived of the medulla oblongata, and exposed to the action of a low temperature, the heart beating, on an average, 35 times in a minute and life lasting four months—*i. e.* 172,800 minutes, it follows that, during that time, the heart has more than 6,000,000 pulsations. In summer, the maximum duration of life having been six hours—*i. e.* 360 minutes, and the heart beating, on an average, 45 times in a minute, it results that, during that life, the heart has only 1,600 pulsations—a number which is to the other as 1 is to 375.

This comparison shows how incorrect it is to suppose that in consequence of the diminution of the vital phenomena in cold weather, a batrachian that lives several months does not live more than another living only several hours in summer.

This opinion is also proved erroneous by the facts which I have already related, and which show that all the functions and vital properties existing in frogs deprived of their medulla oblongata, appear to be as active as in unmutilated frogs. No doubt that there is a notable difference between summer and winter as to the activity of vital phenomena in frogs, but if we suppose that these phenomena are in winter only the tenth of what they are in summer, and if we consider, consequently, the duration of life in winter as being only the tenth of what it is, we shall have, nevertheless, a duration in winter forty or fifty times as great as the duration in summer.

V. Now I have to examine why, in summer, the life of cold-blooded vertebrata, deprived of the medulla oblongata, is much shorter than in winter.

The principal cause of this difference is in the fact that the cutaneous respiration, (it is known that the pulmonary respiration does not exist in animals deprived of the medulla oblongata,) which is sufficient as long as the temperature is very low, becomes more and more insufficient, when the temperature becomes more and more elevated. So that the same law exists for the cold-blooded vertebrata deprived of their medulla oblongata, and for those which are not mutilated. The following experiments concur to demonstrate the correctness of this view:

I have found that frogs deprived of their medulla oblongata live much longer when placed in oxygen than in atmospheric air. I have made two series of experiments—one in June, 1847, the other in July, 1850. In both series the frogs were put immediately after the operation, under a receiver full of oxygen. They lived from eight to fourteen days at a temperature at which life, in atmospheric air, is always shorter than six hours. The temperature was from 64° to 84° F. (18° to 29° Cs.) These frogs would have lived longer if the quantity of oxygen had been more considerable.

By pulmonary insufflation I have maintained life in tortoises deprived of the medulla oblongata, much longer than when insufflation is not used. The duration of life was 7, 12, 13 and 17 days in four insufflated tortoises. When death occurred in these four animals, they had been left without insufflation more than five or six hours, and very likely they would have lived longer had they been insufflated more frequently. In four non-insufflated tortoises life lasted 3, 7, 19 and 23 hours. These comparative experiments were made in summer in a temperature varying from 64° to 86° F. (18° to 30° Cs.) The insufflated tortoises lived longer in summer than non-insufflated tortoises in winter.

From all these facts it is evident that in animals deprived of the medulla oblongata, death is principally caused by insufficiency of respiration.

(To be continued.)

